# Subungual Calcification and Nail Dystrophy: A Case of a Man With Subungual Calcinosis Cutis and an Associated Split Nail Plate

**DOI:** 10.7759/cureus.17695

**Published:** 2021-09-03

**Authors:** Sasha Haddad, Nathan S Uebelhoer, Christof Erickson, Antoanella Calame, Philip R Cohen

**Affiliations:** 1 Dermatology, A.T. Still University, School of Osteopathic Medicine, Mesa, USA; 2 Dermatologic Surgery, San Diego Family Dermatology, National City, USA; 3 Dermatology, Compass Dermatopathology, San Diego, USA; 4 Dermatology/Dermatopathology, Compass Dermatopathology, San Diego, USA; 5 Dermatology, Scripps Memorial Hospital La Jolla, San Diego, USA; 6 Dermatology, University of California, Davis Medical Center, Sacramento, USA

**Keywords:** trauma, thumb, subungual, split, plate, nail, dystrophy, cutis, calcinosis, calcification

## Abstract

A dystrophic nail is an alteration of the physical appearance and structural properties of the nail from its shape, color, and texture, which can result from multiple etiologies. Calcinosis cutis is a condition that refers to the calcium deposition in the skin and underlying tissue. A 55-year-old man who presented with a split right thumbnail associated with subungual calcinosis cutis affecting the same digit for a duration of nine years is described. He did not recall trauma to the affected area. Microscopic evaluation of the tissue specimen obtained following a biopsy of the affected digit’s nail matrix showed foci of calcium in the dermis. The correlation of his clinical presentation and biopsy findings established a diagnosis of subungual calcinosis cutis. Subungual calcification has been observed in several clinical settings. In addition to subungual calcinosis cutis, it has been noted as a normal finding in elderly individuals. In addition, it has been observed in patients with scleroderma or following trauma to the site. Less commonly, subungual calcification can be associated with idiopathic conditions: calcifying aponeurotic fibroma, digital calcinosis circumscripta, subepidermal calcified nodule, and calcified subungual epidermoid inclusion.

## Introduction

The nail is composed of multiple structures that together form the nail unit. The nail unit is surrounded by soft tissue, vessels, and nerves. The nail originates at the matrix and is attached to the nail bed [1,2].

The keratinized nail plate is located on top of the nail matrix and distal portion of the nail bed and bordered by the lateral and proximal nail folds; superficially, the nail plate proximally adheres to the eponychium and distally to the hyponychium. The nail matrix, the proximal portion of the nail bed, is responsible for regenerating a new nail plate; the distal part of the nail matrix can be visualized as a crescent-shaped lunula of the nail bed [3,4].

When the nail matrix, bed, or plate are injured, nail dystrophy can ensue. Dystrophic nails can present with variable morphologic changes that affect the texture, shape, and coloration. Nail plate splitting is a manifestation of a dystrophic nail that develops when the nail plate weakens and becomes brittle; it can also occur following trauma to the nail matrix [3,4].

Calcinosis cutis refers to the deposition of insoluble calcium salts in the skin or the underlying tissue. The five subtypes of calcinosis cutis are calciphylaxis, dystrophic, iatrogenic, idiopathic, and metastatic. Dystrophic calcinosis cutis is the most common type of calcinosis cutis where previously damaged tissue accumulates with insoluble calcium deposition regardless of normal serum calcium and phosphorus level [5].

A man with subungual calcinosis cutis and subsequent nail dystrophy of the same digit is described. Clinical examination revealed a split midline ridge on his right thumbnail; a biopsy of the nail matrix demonstrated calcinosis cutis. The differential diagnosis of subungual calcification is reviewed.

## Case presentation

A 55-year-old man presented for an abnormal asymptomatic right thumbnail of nine years duration. His past medical history was remarkable for hypertension, diabetes mellitus, diabetic neuropathy, hyperthyroidism, hyperlipidemia, anemia, low back pain, left inguinal hernia, pruritus of the skin, psoriasiform dermatitis, obstructive sleep apnea, anxiety, depression. His current medication included glyburide, ibuprofen, lisinopril, metformin, methimazole, and simvastatin.

**Figure 1:**
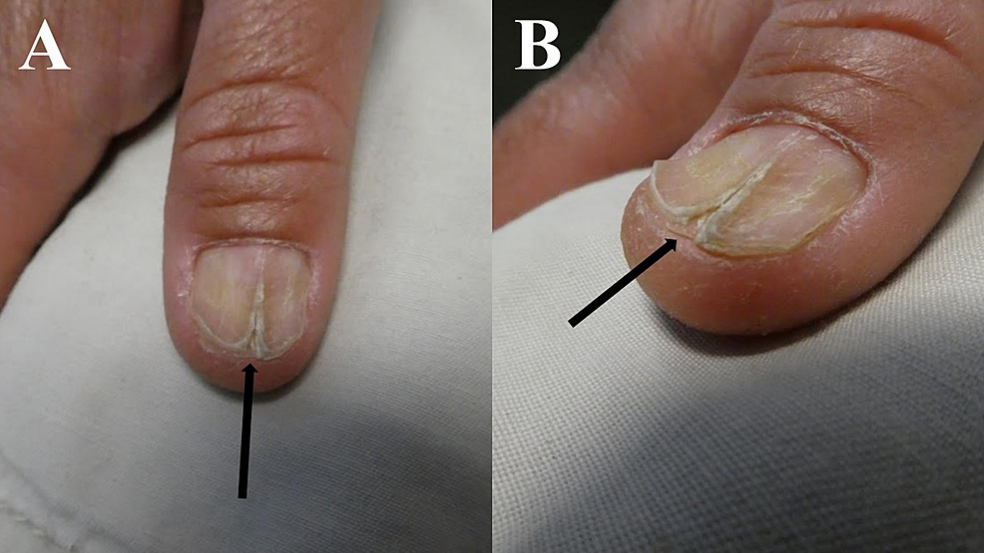
Subungual calcinosis cutis associated linear split of the nail plate of the right thumb Frontal (A) and side (B) views of the right thumbnail of the 55-year-old man demonstrated nail dystrophy characterized by a midline ridge with a split originating distal to the lunula (which represents the portion of the nail matrix that can be visualized) and extending to the tip of the nail plate (black arrow).

Cutaneous examination of the nails on his hands showed a midline ridge with a linear split of the right thumbnail extending from the distal lunula to the end of the nail plate (Figure 1). His other fingernails and toenails were without dystrophy. A roentgenogram of his right thumb did not reveal any abnormalities of the soft tissue or bony structures.

**Figure 2:**
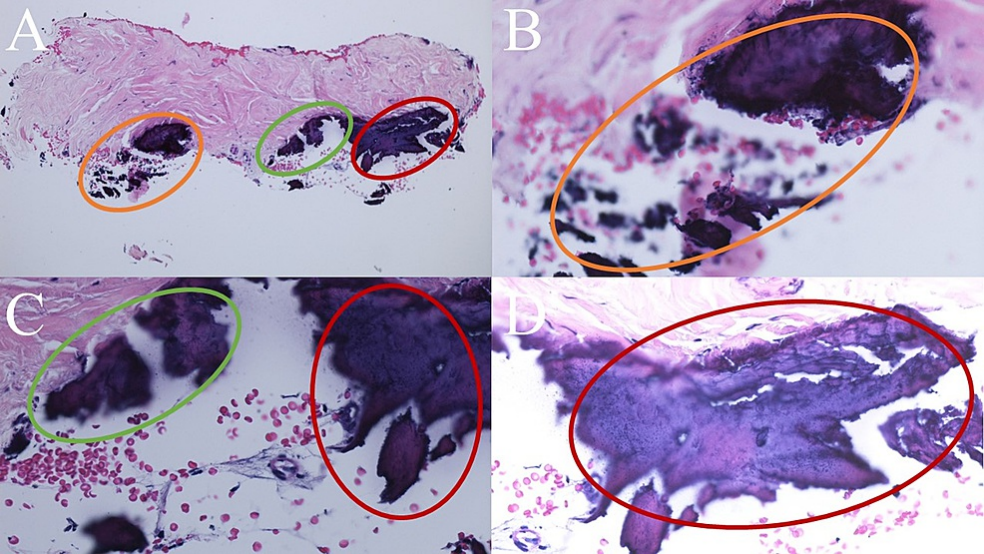
Microscopic features of subungual calcinosis cutis Distant (A) and closer (B, C, and D) views of the tissue specimen from the biopsy of the nail matrix and nail bed show collections of calcium (within the orange, green, and red ovals) in the deep dermis of the nail matrix. The calcification appears as amorphous purple-staining material (Hematoxylin and eosin: A, x2; B, x20; C, x10; D, x20).

A biopsy of the right thumbnail matrix was performed. Microscopic evaluation of the tissue specimen showed islands of basophilic calcium salts present in the sclerotic connective tissue of the dermis (Figure 2). The periodic acid-Schiff (PAS) stain was negative for hyphae.

The pathologic findings were interpreted as calcification within the dermis. Correlation of the clinical presentation and pathology established a diagnosis of subungual calcinosis cutis. His nail dystrophy was attributed to the effect of the calcium present in the nail matrix.

## Discussion

Dystrophic calcinosis cutis occurs in the setting of normal serum calcium and serum phosphorus. It results from local tissue alteration and damage. Although the pathophysiology remains to be definitively established, it has been postulated that necrosed cells release denatured proteins that bind to phosphate molecules and thereafter to calcium followed by a mineralization cascade in an acidic microenvironment [5,6].

Dystrophic calcinosis cutis has been associated with several conditions. These include autoimmune diseases, connective tissue diseases, inherited disorders, miscellaneous conditions, panniculitis, porphyria cutaneous disorder, skin neoplasms, and trauma [5]. Although our patient denied a history of trauma to his thumbnail, we could not identify any non-injury-related etiology for his subungual calcinosis.

In addition to trauma-associated dystrophic calcinosis cutis, several conditions have also been observed in patients with subungual calcification (Table 1) [3-12]. Indeed, elderly individuals have also been found to occasionally have subungual calcification in their normal nail beds. This incidence of subungual calcification in the fingers of older people decreases in frequency from the second to the fifth digit [10].

**Table 1:** Clinical differential diagnosis of subungual calcification CR: current report ^a^These are known as subungual onychodermal cysts. ^b^This has also been referred to as cutaneous calculi.

Condition	Reference
Calcified subungual epidermoid inclusion^a^	[7]
Calcifying aponeurotic fibroma	[4]
Digital calcinosis circumscripta	[8,9]
Normal variant	[10]
Scleroderma	[3,6]
Subepidermal calcinosis nodule^b^	[11,12]
Subungual calcinosis cutis	CR
Trauma	[5,6]

Age-associated subungual calcification has not yet been linked to diseases that have a tendency for the development of tissue calcification. They tend to appear in women as early as the third decade of life with a peak incidence of 47% during the eighth decade. Moreover, these calcifications also appear in elderly men with a smaller incidence of only 14%. The first toe is usually involved up to three times more frequently than the fifth toe [10].

Dystrophic subungual calcification has also been noted in patients with scleroderma, specifically in those affected by calcinosis, Raynaud phenomenon, esophageal dysmotility, sclerodactyly, and telangiectasia (CREST) syndrome. Fingernail changes, calcinosis cutis, ulceration, and microangiopathy have also been observed in patients with more severe forms of systemic sclerosis. In addition to the fingertips, calcinosis cutis also affected the elbows, forearms, hands, metacarpophalangeal joints, and interphalangeal joints [1,5,6].

There are several other, less common, etiologies for subungual calcification. Idiopathic calcinosis cutis can present with subepidermal calcified nodules of the distal digit. These have also been referred to as cutaneous calculi; however, the calcified material in the cutaneous calculi does not necessarily result from the calcification of a pre-existing structure and therefore is not considered to be congenital [11].

A 28-month-old boy with a slowly enlarging solitary congenital nodular calcification of the skin involving the posterior nail wall and the eponychium. The lesion was biopsied and the microscopic examination showed acanthosis, hyperkeratosis, and an irregular mass of calcified granules in the papillary dermis. The investigator attributed the calcification to have occurred secondary to a hamartoma of the sweat glands [12].

Other researchers have collected 10 cases from the literature and described 20 patients with subepidermal calcified nodules that had been seen in Cambridge, England. One of the patients, a 23-year-old man, had a white warty tumor excised from the distal dorsum of his index finger. Microscopy revealed calcified granules that extended into the papillae as well as hyperkeratosis [11].

Individual case reports of subungual calcinosis have also been described in patients with either multiple subungual epidermoid inclusions or calcifying aponeurotic fibroma. A 73-year-old man had an onychodystrophy of the right great toe of seven years duration. He presented with hyperkeratosis of the nail bed and a thick yellow nail plate. After failing antifungal treatment and negative fungal culture, a biopsy of the nail bed revealed multiple calcified subungual epidermoid inclusions in the nail matrix [7].

Calcifying aponeurotic fibroma is a rare benign soft tissue tumor that can present with subungual calcification. It is usually characterized by a painless, firm, and slow-growing lesion that occurs on the distal ends of the extremities; the tumor has areas of calcification, chondrocytes, and fibroblasts. A 44-year-old man was surgically treated for a calcifying aponeurotic fibroma with bone erosion that presented as a swollen nodular mass of two-year duration that was located in the subungual area on the tip of the right index finger; microscopic examination of this tumor showed foci of calcification and proliferated areas of chondrocytes and fibroblasts [4].

Digital calcinosis circumscripta involves the deposition of calcium in the subcutaneous tissue as well as the skin; it specifically occurs on the digits and around joints. An 11-month-old girl with digital calcinosis circumscripta presented with palpable, non-tender subcutaneous nodules on her left third and fourth toe. A radiograph demonstrated cauliflower-like calcifications present in the soft tissue; material from the lesion was diagnosed as calcium phosphate [8].

Similarly, a 9-month-old Latin American female infant presented with a ten-day history of a non-tender, firm white lesion on the distal tip of the second left toe. It was preceded by a month of localized erythema and bumps. The radiopaque mass consisted of fragmented calcifications; subsequently, the patient was diagnosed to have calcinosis cutis circumscripta [9].

Digital calcinosis circumscripta can be differentiated from other types of calcinosis by the extension of the cauliflower-like calcifications towards the skin surface, location at the periphery of the digits, and the ability of the lesion to decrease in size over time. Moreover, calcinosis circumscripta can radiologically mimic tumoral calcinosis. However, the degree of calcification is usually less in calcinosis circumscripta; in addition, the architectural distribution of the calcification is usually in the subcutaneous tissues in calcinosis circumscripta as opposed to bursal regions in tumoral calcinosis [8,9].

No definite treatment has been well described for calcinosis cutis circumscripta but correcting an underlying metabolic derangement has been deemed helpful. Some efforts have focused on limiting further deposition of calcium and reabsorbing existing deposits. In 1929, early investigators proposed a ketogenic diet to help eliminate calcium. Subsequently, a few years later in 1931, other researchers advocated the use of ammonium chloride to change the potential of hydrogen (pH) of the involved tissue fields [8,9].

Unfortunately, neither a ketogenic diet nor ammonium chloride improved calcinosis cutis circumscripta. In addition, drugs such as acetylcholine, calcium disodium edetate, insulin, parathyroid extract, and pilocarpine have all been unsuccessful. However, some researchers advocate removing the lesions, especially when they occur over a joint so that the function of the joint is not hindered [9].

Our patient presented for evaluation of his right thumbnail dystrophy. The biopsy of his nail matrix demonstrated calcinosis cutis. Although he was not able to recall a specific injury to the nail plate or matrix, we suspect he had experienced trauma to the distal digit that resulted in dystrophic calcinosis cutis. Subsequently, the calcinosis cutis affected the nail matrix and caused the split midline ridge of his thumbnail plate.

## Conclusions

Nail dystrophies are often acquired following trauma either to the nail plate or the nail matrix. Various subtypes of calcinosis cutis exist; dystrophic calcinosis cutis occurs at sites of prior tissue injury. Our patient presented with nail dystrophy clinically characterized by a split midline ridge of his right thumbnail; additional investigation revealed calcinosis cutis. We speculate that his subungual calcinosis cutis was associated with a traumatic event to his right thumbnail matrix that he did not recall. Subungual calcification may be observed as an age-related normal variant in elderly individuals. However, it is usually associated with dystrophic calcinosis cutis in patients with benign tumors, autoimmune diseases such as scleroderma, or trauma. Less often, it has been noted in individuals with idiopathic calcinosis cutis presenting as a subepidermal calcified nodule. The diagnosis may not be revealed by a radiograph of the distal finger or toe and may only be established after biopsy of the nail matrix or nailbed or both of the affected digit.
